# Relation between unconjugated bilirubin and RDW, neutrophil to lymphocyte ratio, platelet to lymphocyte ratio in Gilbert’s syndrome

**DOI:** 10.1186/s40064-016-3085-5

**Published:** 2016-08-22

**Authors:** Hakan Sarlak, Erol Arslan, Mustafa Cakar, Mustafa Tanriseven, Salim Ozenc, Muharrem Akhan, Fatih Bulucu

**Affiliations:** 1 Internal Medicine Service, Diyarbakır Military Hospital, Seref Inaloz Street, Yenisehir, 21100 Diyarbakir, Turkey; 2 Department of Internal Medicine, Gulhane Military Medical Faculty, Ankara, Turkey; 3 Department of Rheumatology, Gulhane Military Medical Faculty, Ankara, Turkey; 4 General Surgery Service, Diyarbakır Military Hospital, Diyarbakir, Turkey; 5 Family Medicine Service, Diyarbakır Military Hospital, Diyarbakir, Turkey

**Keywords:** Gilbert’s syndrome, Red cell distribution width, Neutrophil to lymphocyte ratio, Platelet to lymphocyte ratio, Unconjugated bilirubin

## Abstract

**Background:**

Unconjugated bilirubin (UCB) plays a protective role in coronary artery disease. Red cell distribution width (RDW), neutrophil to lymphocyte ratio (NLR) and platelet to lymphocyte ratio (PLR) are inflammatory biomarkers and higher levels are related to atherosclerosis and adverse cardiovascular events.

**Aim:**

We aimed to investigate the relation between UCB levels and RDW, NLR, PLR in people with Gilbert’s syndrome (GS).

**Materials and methods:**

We selected 2166 subjects (1082 with GS and 1084 healthy controls) from a database having 33,695 people. RDW, NLR and PLR were investigated in the subjects with GS and compared with the healthy controls. Linear regression analysis was used to evaluate the relation between variables.

**Results:**

NLR and PLR were higher in the subjects with GS compared to the controls (p < 0.001). RDW was similar in both groups (p = 0.318). UCB was negatively correlated with lymphocyte counts (p = 0.040), and positively correlated with RDW (p < 0.001) and PLR (p = 0.037) in the subjects with GS. There was no significant correlation between UCB and NLR (p = 0.078). RDW (p < 0.001) and lymphocyte counts (p = 0.030) were significantly associated with UCB levels in the regression analysis conducted in the subjects with GS.

**Conclusion:**

There is a negative association between UCB and NLR, PLR due to low amounts of lymphocyte counts, which causes increased risk of CVD. These results suggest that the cardio-protective effect of UCB is due to both anti-oxidative and anti-inflammatory ways indirectly.

## Background

Gilbert’s syndrome is an inborn disorder of bilirubin glucuronidation, characterized by recurrent episodes of jaundice and may be triggered by some conditions such as; dehydration, fasting, menstruation, heavy exercise and other accompanying diseases. As a result of this disorder, serum UCB is above than normal levels (Sieg et al. 1987). The prevalence of GS has been reported to be between 3 and 10 percent in different populations (Kundur et al. 2015). UCB plays a protective role in diseases related with oxidative stress as an antioxidant in low concentrations (20–100 μM) (between 1.17 and 5.85 mg/dL) (Schwertner and Vítek 2008). It has been shown that unconjugated hyperbilirubinemia is associated with decreased risk of coronary and carotid stenosis, peripheral atherosclerosis, ischaemic heart disease, vascular complications in diabetics and even cancer (Vítek et al. 2002; Hjelkrem et al. 2012).

RDW has been reported as an inflammatory biomarker to be increased in myocardial infarction, heart failure, chronic kidney disease and inflammatory status in the literature. Furthermore, it has a prognostic value in patients with heart failure and coronary artery disease (CAD) (Balta et al. 2013a; Cakar et al. 2013). NLR has been shown to be associated with prognosis of malignancies and cardiovascular diseases (Balta et al. 2013b). NLR is rapid and simple parameter of systemic inflammation and related to the severity of CAD (Zahorec 2001; Arbel et al. 2012). A recently published study demonstrated that NLR is an independent predictor of subclinical atherosclerosis in patients with prediabetes (Hamur et al. 2016). High NLR and PLR are related to adverse cardiac events and extent of atherosclerosis (Cicek et al. 2015; Chia et al. 2009; Bian et al. 2010). PLR was introduced as a potential marker to determine inflammation in cardiac and oncologic disorders (Azab et al. 2012; Kwon et al. 2012). NaveenKumar et al. (2015) indicated that UCB induced apoptosis in platelets *in vitro* at the concentration range 0–200 μM (0–11.7 mg/dL). Some studies have demonstrated the association between elevated blood platelet count and major adverse cardiovascular outcomes (Nikolsky et al. 2007; Iijima et al. 2007). Also, Nunez et al. stated that low lymphocyte count was associated with mortality and cardiovascular diseases (Nunez et al. 2011).

In this study, we aimed to investigate the relation between UCB levels and RDW, NLR, PLR in people with Gilbert’s syndrome (GS).

## Methods

### Subjects and data collection

This study was approved by the Local Ethics Committee of Gulhane Military Medical Faculty and designed as a cross-sectional and retrospective study. 2166 subjects were selected among 33,695 people who had applied for a bill of health to our hospital between October 2011 and December 2014 for application of a new job or who wish to continue the existing duty with any known complaint or detected disease (evaluated by the specialists of internal medicine, general surgery, cardiology, urology, ear-nose and throat, eye, orthopedics, neurology, dermatology and psychiatry). Their data were collected from the Military Health Management System (a database used in our hospital). 1082 individuals with the diagnosis of GS and 1084 healthy controls were included in the study. The control group was selected randomly among the people whose all laboratory values were within normal references. All of the GS subjects and controls were males. The viral hepatitis markers, recent drug use history for liver injury were negative and blood pressures were normal in all subjects.

The criteria for the GS diagnosis were: fasting unconjugated bilirubin >1 mg/dL, normal liver function tests, lack of diseases that may cause bilirubin elevation and a normal abdominal ultrasonography (Sieg et al. 1987). The blood samples were collected after an overnight fast. The elevation in UCB at least two different analyses was accepted as GS. Smokers, subjects having a disease that may cause bilirubin elevation and cases having stone in gallbladder or biliary tract were excluded from the study. Due to the small size of the group of individuals over 35 years old, and because they have affected the normal distribution they were excluded from the study. In all subjects age, complete blood count, conjugated bilirubin, unconjugated bilirubin, total bilirubin, alanine aminotransferase (ALT), aspartate aminotransferase (AST), urea, creatinine and fasting plasma glucose levels were recorded.

### Statistical analysis

For statistical analysis, SPSS (Statistical Package for the Social Sciences ver. 15.0, SPSS Inc, Chicago, Illinois, USA) computer program was used. The variables were investigated using visual (histograms, probability plots) and analytical method Kolmogorov–Smirnov test to determine whether or not they are normally distributed. The Pearson correlation test was used to assess the correlation between parametric variables, and the Spearman test was used for nonparametric variables. For comparison of the groups Mann Whitney U/Student’s t-test were used, where appropriate. A multiple linear regression analysis was used to identify independent predictors of UCB. As independent variables age, RDW, platelet and lymphocyte counts were entered into the analysis. Abnormal distributed data was log-transformed for regression analysis. Quantitative variables were expressed as mean ± standard deviation. All statistical tests were 2-tailed and p < 0.05 was considered as statistically significant.

## Results

**Fig. 1:**
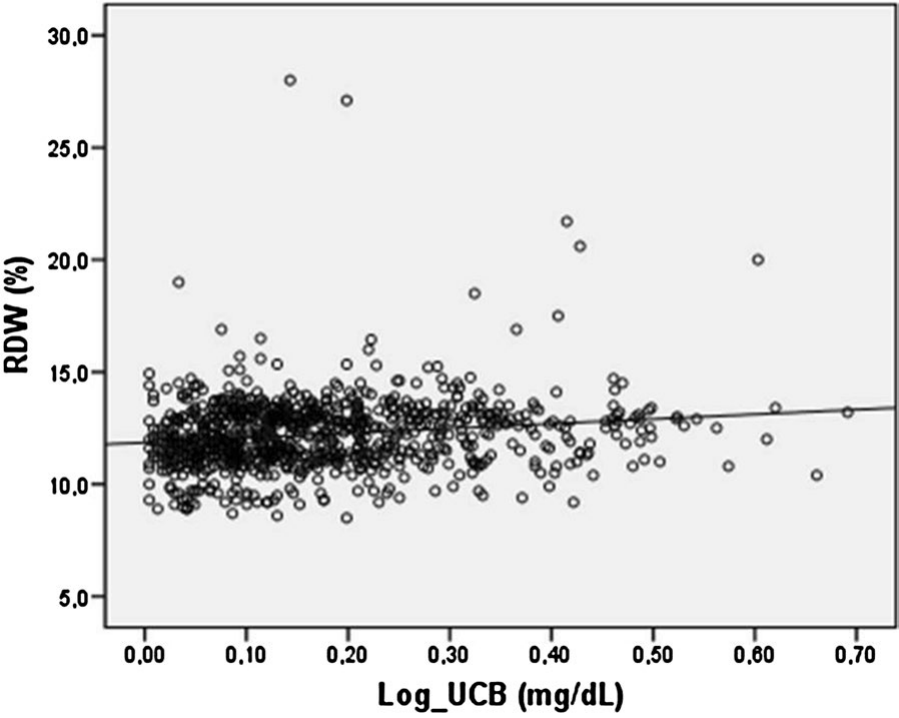
The regression analysis conducted in the subjects with GS (β = 0.181; p < 0.001). UCB was positively correlated with RDW. (UCB was log-transformed for analysis)

**Fig. 2:**
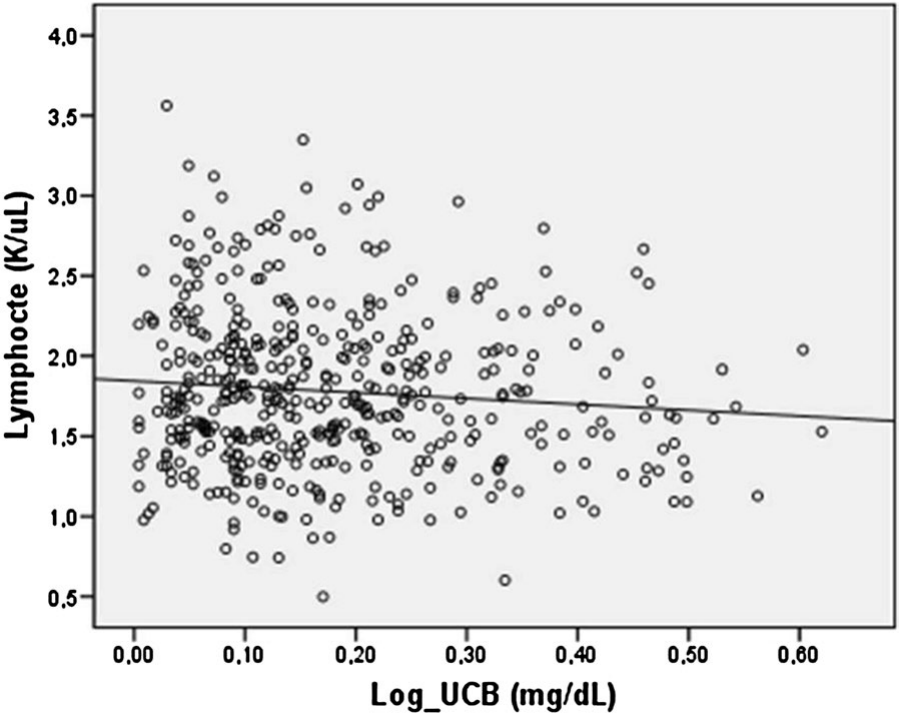
The regression analysis conducted in the subjects with GS (β = −0.100, p = 0.030). UCB was negatively correlated with lymphocyte counts. (UCB was log-transformed for analysis)

The characteristics of the study population were summarized in Table 1. There were 1082 subjects with GS and 1084 healthy controls. The mean ages of the GS and control groups were 22.9 ± 3.6 and 22.8 ± 2.9 years; p = 0.114, respectively (Table 1). The total bilirubin and UCB measurements of the GS and control groups were 1.75 ± 0.53 and 0.59 ± 0.21 mg/dL, 1.56 ± 0.52 and 0.43 ± 0.19 mg/dL; p < 0.001, respectively (Table 1). NLR and PLR levels were higher in the subjects with GS, compared to the controls (2.65 ± 1.05, 1.45 ± 0.50 and 9.95 ± 3.73, 6.80 ± 2.02; p < 0.001, respectively). RDW levels were similar in both groups (p = 0.318). RBC levels were higher in the GS group (p < 0.001). Platelet and lymphocyte counts were lower in the subjects with GS compared to the controls (240.3 ± 53.3, 258.2 ± 55.5 and 1.8 ± 0.5, 2.7 ± 0.6; p < 0.001, respectively). UCB was negatively correlated with lymphocyte and platelet counts, and positively correlated with RBC, urea, creatinine, NLR, neutrophil counts and PLR in the whole group (Table 2). UCB was negatively correlated with lymphocyte counts (Pearson’s r = −0.109, p = 0.040), and positively correlated with RDW (Spearman r = 0.166, p < 0.001) and PLR (Spearman r = 0.098, p = 0.037) in the subjects with GS. There was no significant correlation between UCB levels and NLR (p = 0.078) in the GS group. A multiple linear regression analysis was performed by using the independent variables age, RDW, platelet and lymphocyte counts. RDW (β = 0.181, p < 0.001) and lymphocyte counts (β = −0.100, p = 0.030) were significantly associated with UCB levels in the multiple linear regression analysis conducted in the subjects with GS, respectively (Figs. 1, 2).

**Table 1:** Baseline characteristics of the study population

	Gilbert (n = 1082)	Control (n = 1084)	p value
Age (years)	22.9 ± 3.6	22.8 ± 2.9	.114
Creatinine (mg/dL)	0.97 ± 0.15	0.86 ± 0.12	<.001
Urea (mg/dL)	28.5 ± 7.3	27.6 ± 6.0	.158
Fasting glucose (mg/dL)	86.6 ± 11.3	85.4 ± 8.4	.040
UCB (mg/dL)	1.56 ± 0.52	0.43 ± 0.19	<.001
TB (mg/dL	1.75 ± 0.53	0.59 ± 0.21	<.001
Hb (g/dL)	15.8 ± 1.2	15.3 ± 1.0	<.001
RBC (K/μL)	5.44 ± 0.44	5.17 ± 0.38	<.001
Plt (K/μL)	240.3 ± 53.3	258.2 ± 55.5	<.001
RDW (%)	12.2 ± 1.6	12.3 ± 0.8	.318
WBC (K/μL)	7.2 ± 2.0	7.0 ± 1.3	.741
Lymphocyte (K/μL)	1.8 ± 0.5	2.7 ± 0.6	<.001
Neutrophil (K/μL)	4.4 ± 1.4	3.8 ± 1.1	<.001
NLR	2.65 ± 1.05	1.45 ± 0.50	<.001
PLR	9.95 ± 3.73	6.80 ± 2.02	<.001

*UCB* unconjugated bilirubin, *TB* total bilirubin, *Hb* hemoglobin, *RBC* red blood cell, *Plt* platelets, *RDW* red cell distribution width, *WBC* white blood cell, *NLR* neutrophil to lymphocyte ratio, *PLR* platelet to lymphocyte ratio. Independent samples t test for normally distributed data. Mann Whitney U test for abnormally distributed data. Values are given as mean ± standard deviation

**Table 2:** Correlations in the whole group

	UCB (mg/dL)
RBC (K/μL)	
r	0.252
p	<0.001
Lymphocyte (K/μL)	
r	−0.542
p	<0.001
Urea (mg/dL)	
r	0.063
p	0.004
Creatinine (mg/dL)	
r	0.317
p	<0.001
Platelet (K/μL)	
r	−0.149
p	<0.001
NLR	
r	0.489
p	<0.001
Neutrophil (K/μL)	
r	0.150
p	<0.001
PLR	
r	0.357
p	<0.001

*UCB* unconjugated bilirubin, *RBC* red blood cell, *NLR* neutrophil to lymphocyte ratio, *PLR* platelet to lymphocyte ratio. Pearson correlation analysis for normally distributed data. Spearman’s correlation analysis for abnormally distributed data

## Discussion

RDW, NLR and PLR are the predictors of subclinical atherosclerosis at higher levels. In our study we aimed to investigate the relation between these markers and UCB levels. Furthermore, we aimed to determine whether there is an association between UCB levels with these inflammatory markers. In the present study we found that UCB was associated with RDW and PLR in GS group. There was no association between UCB and NLR. In GS group higher NLR and PLR might be due to the lower amounts of lymphocytes, compared to neutrophil and platelet counts.

In the present study UCB was significantly and positively associated with RDW levels in regression analysis conducted in the GS group. RDW as a marker of the variability in erythrocyte volume is a routinely available component of the complete blood count (CBC). In patients with ineffective red cell production, increased red cell destruction and blood transfusion, RDW levels can be elevated (Förhécz et al. 2009). RDW reflects variability in the size of circulating red cells and is routinely reported by analyzers as part of routine CBCs. Several studies in the literature have reported that elevated RDW levels are significantly associated with a poor prognosis in the setting of heart failure, acute myocardial infarction and stable angina pectoris. RDW is recognized as a global marker of chronic inflammation and oxidative stress (Balta et al. 2013a; Cakar et al. 2013). However, the underlying biological mechanisms remain unclear. In our study the association between UCB and RDW might only be the response of bone marrow to increased red cell destruction in the subjects with GS and so RDW does not reflect the inflammation.

In our study RBC levels were higher in the subjects with GS compared to the healthy controls. Wallner et al. showed a positive correlation between unconjugated bilirubin and free heme, iron and carboxy hemoglobin which might be the result of a positive feedback loop of heme oxygenase induction mediated by unconjugated bilirubin (Wallner et al. 2013). Similarly, bone marrow can be stimulated by high amounts of iron, free heme and carboxy hemoglobin which have been released from the hemolytic process in the subjects with GS in our study. Therefore, because of the activation of bone marrow the RBC levels might be higher in the GS group.

Platelet counts were significantly lower in the subjects with GS compared to the control group in our study. Suvansri et al. (1969) showed that UCB affected platelet volume and platelet count in hyperbilirubinemic individuals. NaveenKumar et al. found that UCB induced apoptotic events in platelets including elevated endogenous reactive oxygen species (ROS) generation, mitochondrial membrane depolarization, increased intracellular calcium levels, cardiolipin peroxidation and phosphatidylserine externalization. They also indicated that elevated UCB caused thrombocytopenia by stimulating platelet apoptosis via mitochondrial ROS-induced p38 and p53 activation (NaveenKumar et al. 2015). This may be one of the main mechanisms which cause a reduction in the platelet counts in our study. Moreover, lower platelet counts cause lower P-selectin levels, which leads to less thrombotic events (Kundur et al. 2015). P-selectin is an adhesion molecule released from granules of activated platelets and endothelial cells. It mediates the adhesion of platelets and inflammatory cells to the endothelial surface (Blann and Lip 1997; McEver 1991). Tapan et al. (2009) showed decreased soluble P-selectin levels in GS. P-selectin can attract inflammatory cells, induce endothelial cell activation and platelet aggregation when released from activated platelets (Blann and Lip 1997; Lievens et al. 2010). Also, P-selectin plays an essential role in thrombus formation by facilitating the development of large stable platelet-leukocyte aggregates (Yokoyama et al. 2005). Ridker et al. (2001) demonstrated that healthy individuals with elevated concentrations of P-selectin have increased risk of suffering a future cardiovascular event. Moreover, inhibition of circulating P-selectin is associated with a reduced risk of thrombus formation and is evolving as a therapeutic target for treatment and prevention of future CVD (Chelliah et al. 2009). These findings suggest that GS subjects may have lower risk of thrombosis due to an indirect effect of bilirubin reducing P-selectin levels which leads to less cardiovascular events.

NLR may act as a marker of inflammation in several cardiac and non-cardiac conditions (Balta et al. 2016). Hamur et al. (2016) demonstrated that higher NLR and lower total bilirubin levels were independent predictors of subclinical atherosclerosis in patients with prediabetes. They showed a negative correlation between NLR and total bilirubin in their study. Conversely there was a positive correlation between total bilirubin and NLR in our study. However, in their study total and unconjugated bilirubin levels were within normal ranges. It would be wrong to make an inference with normal values of bilirubin. We hypothesize that UCB has a lowering effect on lymphocytes in mildly elevated levels. The NLR was higher in GS group compared to controls because of the low lymphocyte counts in this group. Therefore a positive correlation between UCB and NLR was observed in our study.

In our study we found significantly lower lymphocyte counts in the GS group compared to the control group. UCB has been shown to lead to apoptosis of cells through an association of UCB with mitochondrial membranes, leading to cytochrome C release into the cytosol and activation of caspase-9 (Keshavan and Schwemberger 2004). This condition may be the result of apoptotic effect of UCB lowering lymphocyte counts (Jangi et al. 2013).

### Study limitations

This study aims to use the advantage of a huge number of GS subjects on complete blood cell count parameters. But as a retrospective and cross-sectional study, our study lacks the benefit of long time prospective case control studies. Our findings are inherently limited in the ability to eliminate the causal relationships between RDW, NLR, PLR and bilirubin. Higher ratios of NLR and PLR are due to low lymphocyte counts in the GS group in our study. Therefore, the interpretation of NLR and PLR are difficult, because lymphocyte counts are decreased. Additional markers of neutrophil and platelet activation are required (i.e. P-selectin, L-selectin) to determine the significance of these findings. Further prospective population based studies are needed to investigate the mechanisms in order to answer these questions. Determining the levels of inflammatory markers in serum samples of GS subjects in terms of endothelial dysfunction or measuring the flow mediated dilation or arterial stiffness of these subjects would give information beyond using only complete blood count parameters, but these measurements were not practical for so many people. Lack of lipid profile, uric acid levels, other inflammatory markers such as high sensitive C-reactive protein and BMIs restricted our comments about inflammatory properties of UCB in the subjects. Other limitations are that only males and under the age of 35 years were included in this study. Complete blood cell parameters may be affected by some factors such as the waiting time of blood sample in tubes before laboratory study or an unannounced inflammatory condition. This may have caused some disturbances in our results.

## Conclusion

In this study we found that there was a reduction in platelet counts in mildly elevated (1–4 mg/dL) serum levels of UCB. The ongoing apoptotic effect of UCB on platelets has been shown in other studies at higher levels to 11.7 mg/dL. We believe that, UCB has a protective effect on CVD indirectly by affecting the pro-inflammatory processes. However, we saw that there is a negative association between UCB and NLR, PLR due to low amounts of lymphocyte counts, which causes increased risk of CVD. These results suggest that the cardio-protective effect of UCB is due to both anti-oxidative and anti-inflammatory ways indirectly.
